# Advanced therapy options, choice, access, and treatment empowerment for inflammatory bowel disease (ADVOCATE-IBD): a UK-wide semi-structured interview study exploring patient preferences around advanced therapy decisions in inflammatory bowel disease

**DOI:** 10.1093/crocol/otag098

**Published:** 2026-08-28

**Authors:** Ronit Hardasani, Shellie Jean Radford, Mohammad Farhad Peerally

**Affiliations:** School of Medicine, University of Leicester, Leicester LE1 7RH, United Kingdom; NIHR Nottingham Biomedical Research Centre, Nottingham NG7 2UH, United Kingdom; SAPPHIRE, Division of Public Health and Epidemiology, University of Leicester, Leicester LE1 7RH, United Kingdom; Digestive Disease Unit, Kettering General Hospital, University Hospitals of Northamptonshire NHS Group, Kettering NN16 8UZ, United Kingdom

**Keywords:** inflammatory bowel disease, patient preference, qualitative, interviews, shared decision making, biologics

## Abstract

**Background:**

Advanced therapies, including biologics and small molecule drugs, are increasingly used in the management of inflammatory bowel disease (IBD), but their diverse characteristics can influence patient treatment choices and experiences. Understanding patient priorities and practicing shared decision-making can optimize treatment satisfaction and adherence. This qualitative study explored patient experiences on advanced therapies to identify factors influencing treatment decisions aiming to develop a practical tool supporting shared decision-making when initiating or switching treatments.

**Method:**

Using purposive sampling, participants on advanced therapies for moderate-to-severe IBD were recruited through Crohn’s & Colitis UK and social media until achieving data saturation. Semi-structured interviews were conducted using a topic guide developed from literature review and patient representative input. Audio recordings were transcribed and analyzed using reflexive thematic analysis.

**Results:**

Of 26 participants, most were female (73.1%) and using Vedolizumab (30.7%) or Infliximab (26.9%). The age group 31-40 (34.6%) was most commonly represented, and 46.2% had ulcerative colitis. All participants were in self-reported clinical remission. Four overarching themes were identified: embedding treatment realities into daily routines; negotiating the tension between therapeutic benefits and potential risks; navigating formal and informal support structures; and asserting agency in treatment decisions.

**Conclusion:**

Patient decision-making around advanced therapies for the management of moderate-to-severe IBD is influenced by treatment practicality and life impact, balancing efficacy and risk management, access to healthcare support networks, and involvement in care decisions. The Advanced therapy options, choice, access, and treatment empowerment for IBD tool, developed from these findings, provides clinicians with a framework to understanding individual patient priorities and values, supporting meaningful shared decision-making.

## Graphical Abstract

**Graphical Abstract:**
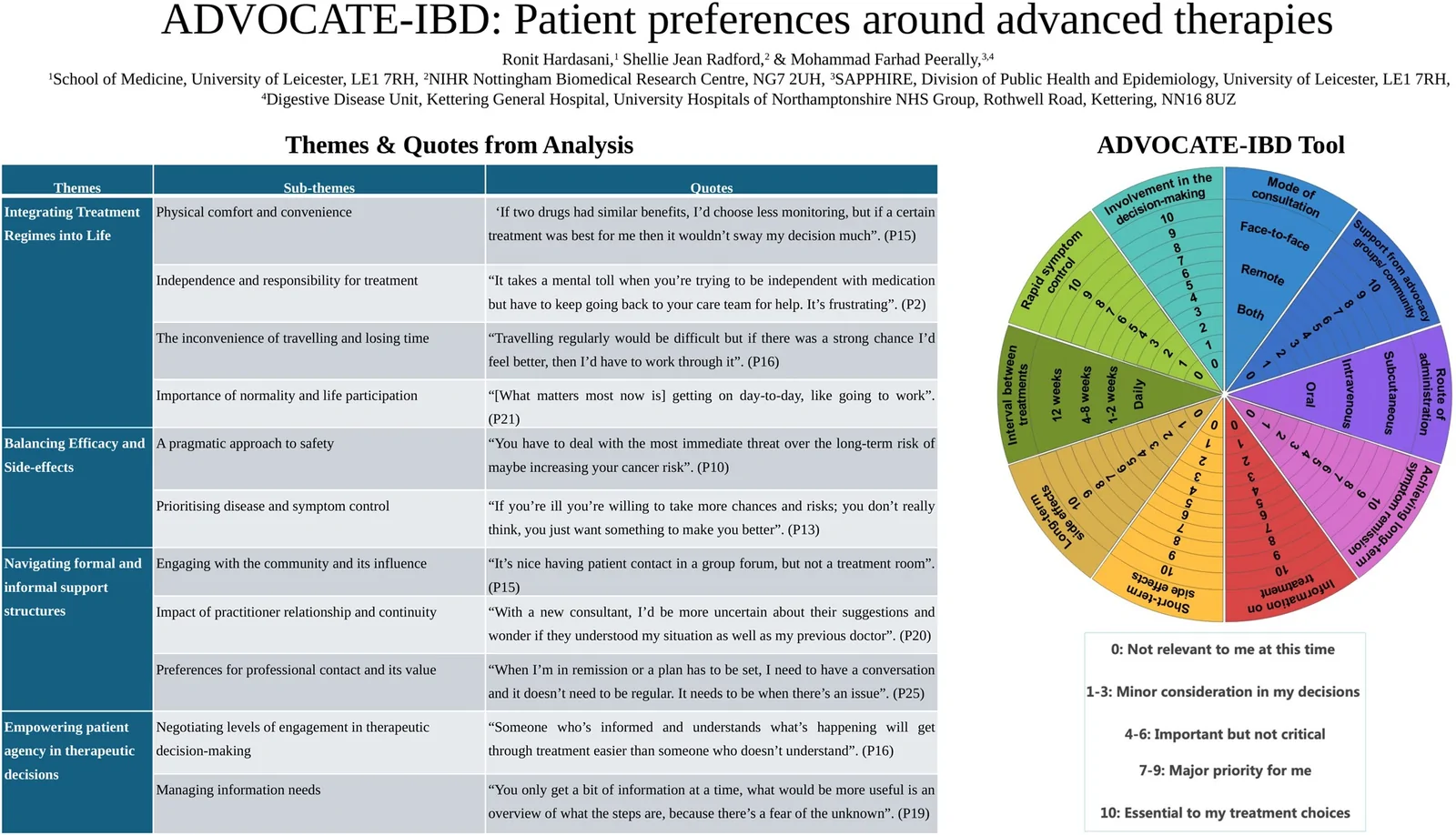

## Introduction

Inflammatory bowel disease (IBD) refers to a group of chronic conditions, including Crohn’s disease (CD) and ulcerative colitis (UC), that impact almost 7 million individuals worldwide,^1^ with peaks in incidence between ages 20-30 and 50-70.^2^^,^^3^ The lived experience of IBD manifests through symptoms including abdominal pain, fatigue, rectal bleeding and fecal incontinence, profoundly impacting health-related quality of life.^4^

Contemporary care pathways, delivered through multi-disciplinary teams, increasingly utilize targeted biological agents and small molecule drugs, collectively termed “advanced therapies” for the management of moderate-to-severe IBD.^5^ These therapeutic interventions, available through varied routes of administration, target specific inflammatory pathways to modify disease progression. Their therapeutic impact extends beyond clinical efficacy, encompassing various dimensions of treatment burden including safety profiles, monitoring requirements, and administration modalities.

The expanding therapeutic landscape offers opportunities for treatment individualization, potentially enhancing patient engagement with care regimens. Tailoring treatment to reflect individual values enhances patient satisfaction and adherence, improving long-term health outcomes such as risks of complications, disease relapse or progression.^6^^,^^7^ However, reported medication adherence rates of 55%-70% in patients with IBD indicate persistent challenges in treatment optimization.^8^^,^^9^ Potential reasons may include underlying tensions between clinician and patient priorities, and insufficient recognition of the lived experience of IBD within healthcare systems.^10^^,^^11^

By incorporating shared decision-making practices, healthcare professionals can better understand individual priorities and reasoning, promoting patient-centered care and optimize health outcomes.

Previous studies show that patients factor symptom control,^12–14^ professional guidance,^15–17^ route of drug delivery,^12^^,^^17^^,^^18^ and serious side effects,^2^^,^^18^^,^^19^ into their decision-making regarding therapies for IBD. However, there remains limited qualitative understanding of what patients with moderate-to-severe IBD consider as important factors influencing their choices of advanced therapies. This gap in knowledge limits our understanding of how patients construct meaning around treatment decisions and engage with increasingly complex therapeutic options.

This study therefore aimed to explore patient experiences regarding treatment with advanced therapies for moderate-to-severe IBD through 2 key questions: “What factors are important to patients when starting advanced therapies for IBD?” and “How do patients define and experience quality care while managing their IBD with advanced therapies?”. Through this exploration, we sought to develop a practical tool to support shared decision-making in clinical consultations, accounting for individual preferences and values around advanced therapies.

## Methods

### Recruitment and sampling

Participants were recruited through multiple complementary channels to enhance sample diversity and mitigate selection bias. First, we collaborated with the patient advocacy group Crohn’s & Colitis UK (CCUK), who disseminated study information through newsletters and targeted mailing lists. Second, we implemented a social media outreach strategy using Facebook and X (formerly Twitter), creating dedicated recruitment posts that were shared across IBD-focused community groups. Third, we utilized professional networks to raise study awareness, with gastroenterologists and IBD nurses, known to the investigators, directing potential participants to our study webpage where they could access information independently. No incentives were offered.

Participants were sampled purposively based on age, sex, geographical locations, medications, underlying conditions and duration on therapy, to select those likely to provide “rich” responses, capturing nuanced insights that randomized sampling may overlook.^20^ We intentionally recruited participants with a range of duration since diagnosis to capture perspectives across the therapeutic journey.

This approach enabled us to understand how preferences and priorities evolve over time with treatment experience, providing a more comprehensive view of factors influencing sustained engagement with advanced therapies rather than only initial decision-making considerations. Eligible participants were UK residents aged 18 or older, taking advanced therapies for IBD and able to communicate in English. Individuals who responded to recruitment posts were given information about the study, and after at least 48 hours were sent a consent form to sign. Ethical approval was received from the University of Leicester Medicine and Biologic Sciences Research Ethics Committee (approval number 42484).

### Data collection and analysis

Each participant engaged in a 60-minute semi-structured interview via Microsoft Teams^TM^. All interviews were conducted by a single researcher (author A), with regular team discussions to reflect on findings during the data analysis phase. While this ensured consistency in the interview approach, we acknowledge the potential for interviewer bias. To mitigate this, the analysis involved multiple investigators (authors A-C) who collaboratively developed the thematic framework. Interviews were structured around a topic guide (see Online SupplementaryAppendix 1), designed using peer-reviewed literature and input from patient-representatives, but flexible to adaptation as analytically constructed insights developed. This topic guide explored patient experiences and preferences around treatment and monitoring, factors influencing decision-making, and their perspectives on what constitutes high-quality care while receiving advanced therapies. Additionally, a patient and public involvement (PPI) group of 4 patients, diverse in gender, ethnicity and IBD classification, provided feedback on study design. PPI members did not participate in data collection, analysis, or interpretation.

Interviews continued until reaching data saturation, defined by the absence of analytically novel codes arising from concurrent analysis. Sample size in qualitative interview research is appropriately guided by data saturation—the point in data collection when no new insights are identified leading to a repetition in findings drawing data collection to an end—rather than by statistical power calculations applied in quantitative paradigms.^21^ Audio-recordings were transcribed verbatim, anonymized and re-read for familiarization. Key concepts were then coded using NVivo 12, QSR International, Australia. Codes were discussed regularly with authors B and C before being aggregated into over-arching themes through reflexive thematic analysis.^22^

This iterative approach allowed codes to continuously evolve while recognizing the influence of researcher experiences on data interpretation. Author A undertook formal training on qualitative methods and authors B and C are established qualitative clinical researchers. This study was conducted and reported using the Standards for Reporting Qualitative Research (SRQR) (Online Supplementary Appendix 2) guidelines to ensure transparency and rigor.

### Designing the ADVOCATE-IBD tool

Following thematic analysis, we identified key domains that were analytically developed and constructed as important to patients when considering advanced therapies. These domains were translated into a visual representation modeled after the validated IBD-Disk^23^ format to enhance clinical utility. Each included domain corresponds directly to a major theme or subtheme from our analysis (Table 1), ensuring the framework authentically represents patient-identified priorities.

**Table 1:** Themes/subthemes corresponding to domains in ADVOCATE-IBD tool.

Theme/subtheme from analysis	Domain
**Negotiating levels of engagement in therapeutic decision-making**	Involvement in the decision-making
**Preferences for professional contact and its value**	Mode of consultation
**Engaging with the community and its influence**	Support from advocacy groups/community
**Physical comfort and convenience**	Route of administration
**Prioritizing disease and symptom control**	Achieving long-term symptom remission
**Managing information needs**	Information on treatment
**A pragmatic approach to safety**	Short-term side effects
**A pragmatic approach to safety**	Long-term side effects
**Importance of normality and life participation**	Interval between treatments
**Prioritizing disease and symptom control**	Rapid symptom control

## Results

### Participant characteristics

Between February and May 2024, 71 potential participants reached out to the research team by email, of whom 42 consented to participating. A total of 26 interviews were conducted, averaging a duration of 61 minutes. Participants were mostly female (73.1%), 46.2% employed full-time, and the most commonly represented age group was 31-40 (34.6%). There was an equal proportion of participants with CD and UC (46.2%), of whom the majority had been living with a diagnosis of IBD for more than 5 years (65.3%). Patients were on different biologics and small molecules, as shown in Table 2, with Vedolizumab (30.7%) and Infliximab (26.9%) being the most commonly used advanced therapies. Importantly, all 26 participants were in clinical remission at the time of interview, defined as patient-reported absence of active symptoms. Participants had experienced varying numbers of prior advanced therapies before achieving remission on their current treatment: 11 participants (42.3%) achieved remission on their first advanced therapy, 4 (15.4%) had tried 1 previous therapy, 6 (23.1%) had tried 2, 2 (7.7%) had tried 3, 1 (3.8%) had tried 4, and 1 (3.8%) had tried 5 previous advanced therapies; notably, 1 participant achieved remission on a combination of subcutaneous Ustekinumab and intravenous Vedolizumab following failure of 2 prior biologics. The participants’ baseline characteristics are shown in Table 2.

**Table 2:** Participant baseline characteristics.

Characteristic	Number of participants, *n* (%)
**Sex**	
**Male**	7 (26.9)
**Female**	19 (73.1)
**Age range**	
**18-20**	1 (3.8)
**21-30**	5 (19.2)
**31-40**	9 (34.6)
**41-50**	5 (19.2)
**51-60**	3 (11.5)
**61-70**	2 (7.7)
**71-80**	1 (3.8)
**Inflammatory bowel disease classification**	
**Ulcerative colitis**	12 (46.2)
**Crohn’s disease**	12 (46.2)
**Unclassified**	2 (7.7)
**Time since diagnosis**	
**≤1 year**	2 (7.7)
**1-5 years**	7 (26.9)
**5-10 years**	11 (42.3)
**10-20 years**	3 (11.5)
**>20 years**	3 (11.5)
**Current advanced therapy**	
**Adalimumab**	5 (19.2)
**Filgotinib**	1 (3.8)
**Infliximab**	7 (26.9)
**Upadacitinib**	3 (11.5)
**Ustekinumab**	3^a^ (11.5)
**Vedolizumab**	8^a^ (30.7)
**Current route of administration**	
**Oral**	4 (15.3)
**Subcutaneous injection**	13^a^ (50)
**Intravenous infusion**	10^a^ (38.4)
**Time on current advanced therapy**	
**≤6 months**	6 (23)
**6 months-1 year**	7 (26.9)
**1-5 years**	11 (42.3)
**5-10 years**	2 (7.7)
**Region of care**	
**Northumberland**	1 (3.8)
**Bedfordshire**	1 (3.8)
**Belfast**	1 (3.8)
**Greater Manchester**	1 (3.8)
**Cornwall**	1 (3.8)
**Derbyshire**	1 (3.8)
**West Midlands**	1 (3.8)
**Surrey**	2 (7.7)
**Leicestershire**	5 (19.2)
**Norfolk**	1 (3.8)
**London**	4 (15.3)
**Nottinghamshire**	2 (7.7)
**Hampshire**	2 (7.7)
**Yorkshire**	3 (11.5)
**Occupational status**	
**Unemployed**	4 (15.3)
**Student**	2 (7.7)
**Employed full-time**	12 (46.2)
**Employed part-time**	5 (19.2)
**Retired**	1 (3.8)
**Self-employed**	2 (7.7)
**Ethnicity**	
**White British**	18
**Greek**	1
**Croatian**	1
**Italian**	1
**South Asian**	4
**Chinese**	1

a Participant taking both Intravenous Vedolizumab and Subcutaneous Ustekinumab.

### Patient preferences and values

Four themes were identified which influenced patient decision-making and constituted high-quality care: integrating treatment regimens into life; balancing efficacy and side-effects; navigating formal and informal support structures; empowering patient agency in therapeutic decisions. These are shown in Table 3, along with subthemes and illustrative quotes.

**Table 3:** Overview of themes with illustrative quotes.

Themes	Sub-themes	Quotes
**Integrating treatment regimes into life**	Physical comfort and convenience	“I didn’t like the idea of having to inject but did get used to it eventually.” (P1) “If two drugs had similar benefits, I’d choose less monitoring, but if a certain treatment was best for me then it wouldn’t sway my decision much.” (P15)
	Independence and responsibility for treatment	“It takes a mental toll when you’re trying to be independent with medication but have to keep going back to your care team for help. It’s frustrating.” (P2)
	The inconvenience of traveling and losing time	“Travelling regularly would be difficult but if there was a strong chance I’d feel better, then I’d have to work through it.” (P16)
	Importance of normality and life participation	“[What matters most now is] getting on day-to-day, like going to work.” (P21)
**Balancing efficacy and side-effects**	A pragmatic approach to safety	“You have to deal with the most immediate threat over the long-term risk of maybe increasing your cancer risk.” (P10) “Reading the side-effects can give you a shock but you have to keep the mindset that it’s only a very small chance.” (P16)
	Prioritizing disease and symptom control	“If you’re ill you’re willing to take more chances and risks; you don’t really think, you just want something to make you better.” (P13)
**Navigating formal and informal support structures**	Engaging with the community and its influence	“It’s nice having patient contact in a group forum, but not a treatment room.” (P15) “I found the community quite negative and didn’t want to be surrounded by people who more ill than myself.” (P23)
	Impact of practitioner relationship and continuity	“With a new consultant, I’d be more uncertain about their suggestions and wonder if they understood my situation as well as my previous doctor.” (P20)
	Preferences for professional contact and its value	“When I’m in remission or a plan has to be set, I need to have a conversation and it doesn’t need to be regular. It needs to be when there’s an issue.” (P25)
**Empowering patient agency in therapeutic decisions**	Negotiating levels of engagement in therapeutic decision-making	“Someone who’s informed and understands what’s happening will get through treatment easier than someone who doesn’t understand.” (P16) “[Professionals can] present different options, saying what they would do then ultimately let patients choose.” (P24)
	Managing information needs	“You only get a bit of information at a time, what would be more useful is an overview of what the steps are, because there’s a fear of the unknown.” (P19)

### Integrating treatment regimes into life

The experience of drug administration and their varied associated temporal and logistical demands were identified as important factors in therapeutic decision-making among patients with IBD. Route of administration preferences were dynamically shaped by physical comfort and practical considerations. Tablets were “easily built into a routine” *(P13)*, in contrast to self-injecting as “every 14 days isn’t routine” *(P26)*. In comparison, those on infusions felt “tethered to hospitals” *(P24)*, due to the duration of treatments and post-infusion fatigue “making working impossible” *(P11)*. The low frequency of infusions was valuable to patients wanting to “forget [their] IBD” *(P10)* as feeling normal was a key goal. This duality between therapeutic necessity and life participation, deemed “difficult without treatment” *(P15),* were identified consistently across accounts, highlighting how treatments had to be embedded within broader life contexts including work, social relationships, and daily routines.

The tension between maintaining autonomy and accepting the responsibilities of self-administered therapy was another important factor in treatment decisions. While hospital-based infusions provided a supervised environment, many participants valued the self-determination offered by home administration, despite initial hesitation: “I quickly realised it wouldn’t be problematic. The idea was more concerning than actually doing it” *(P23)*. This reflects the complex negotiation between independence and the practical demands of managing chronic illness.

The monitoring burden of the disease and the advanced therapies represented a notable aspect of the illness experience, particularly regarding endoscopic surveillance. While blood tests and other monitoring became normalized over time as part of the illness trajectory, endoscopic procedures remained particularly challenging. Patients consistently showed a desire for alternatives to endoscopies, often described as “traumatic” *(P12)* due to bowel preparation and their invasive nature, expressing a desire for less intrusive and less frequent monitoring strategies.

### Balancing efficacy and side-effects

The complex interplay between treatment efficacy and safety considerations was a key theme in participants’ therapeutic journeys. While long-term safety concerns persisted, particularly regarding risk of malignancy “[we could] get cancer, either from [uncontrolled] IBD or the drugs,” participants often prioritized immediate disease control: “You have to deal with the most immediate threat over the long-term risk of maybe increasing your cancer risk” *(P10)*. This pragmatic approach was echoed in attitudes toward general safety concerns, with participants expressing that there was “no point worrying about stuff [they] can’t control” *(P2)*.

The evaluation of potential adverse effects in fact revealed a nuanced risk assessment process. While participants acknowledged the intimidating nature of safety information, they developed coping strategies: “Reading the side-effects can give you a shock but you have to keep the mindset that it’s only a very small chance” *(P16)*. Furthermore, many participants had never experienced an adverse event, so did not consider them “unless [they] got any” *(P5)*, hoping to avoid being discouraged from potentially effective treatments.

Disease severity significantly influenced risk tolerance, with acute symptoms driving more urgent treatment decisions: “If you’re ill you’re willing to take more chances and risks; you don’t really think, you just want something to make you better” *(P13)*. This prioritization of symptom control was particularly evident among acutely unwell patients who “prioritised feeling better soon” *(P20)*, while those with controlled disease focused more on maintaining remission. Additional therapeutic considerations included immunogenicity, demonstrating good knowledge of therapeutic mechanisms of action by certain patients who expressed concern about “creating antibodies” *(P18),* though some accepted this as “something [they] couldn’t avoid” *(P6)*.

### Navigating formal and informal support structures

The social ecology of peer and professional therapeutic support was a valuable aspect of participants’ experiences of care when on advanced therapies. The role of peer networks demonstrated notable complexity, with participants strategically managing their engagement with the IBD community. While peer support was valued in the early stages of therapeutic journeys, participants’ preferences for engagement varied contextually: “It’s nice having patient contact in a group forum, but not a treatment room” *(P15)*. This selective engagement reflected individualized illness management strategies, with some finding inspiration in others’ success stories while others actively distancing themselves from collective illness narratives, noting resistance to being “surrounded by people sicker than [themselves]” *(P23)*, particularly during treatment administration. Despite some participants considering the community as being “quite negative,” and “not [wanting to] talk about being ill all the time,” *(P23)*, online peer support was preferred over face-to-face interactions during infusions, as many patients “went for treatment, keeping to themselves” *(P24)*.

The therapeutic alliance between patients and healthcare providers was viewed by participants as crucial, with continuity of care influencing treatment confidence: “With a new consultant, I’d be more uncertain about their suggestions and wonder if they understood my situation as well as my previous doctor” *(P20)*. The quality of this relationship was enhanced when clinicians recognized patients’ experiential knowledge and autonomy in “treating their [own] disease” *(P13)*, while continuity reduced the emotional burden of repeatedly “re-explain[ing] the things you do anyway in life” *(P24)*.

Preferences for engagement with healthcare services varied depending on disease activity and individual needs: “When I’m in remission or a plan has to be set, I need to have a conversation and it doesn’t need to be regular. It needs to be when there’s an issue” *(P25)*. IBD specialist nursing services were an important support mechanism, with participants particularly valuing the accessibility of immediate professional guidance through helplines, knowing they “could call for immediate advice” *(P15)*. For consultant follow-ups, some favored ad-hoc regimes although biannual appointments were often sufficient. Patients were also confident that IBD nurses offered high-quality care, considering 3-to-6 monthly appointments with them to be adequate. Although face-to-face interactions offered benefits through “facial expressions and body language” *(P22)*, the convenience of remote consultations often took precedence, highlighting the balance between therapeutic value and practical accessibility.

### Empowering agency in therapeutic decision-making

While some patients fully trusted a care provider-led approach and were “happy just being told what’s happening” *(P22)*, many sought an active role in their care in case “what [they] said changed doctors’ decisions” *(P19)*. This engagement was viewed as fundamental to treatment success: “Someone who’s informed and understands what’s happening will get through treatment easier than someone who doesn’t understand” *(P16*).

The expression of patient agency demonstrated notable variation across the illness trajectory, with particular challenges during acute disease phases or early post-diagnosis periods. During these vulnerable periods, participants often felt disempowered by being “with no treatment knowledge or anyone to ask” *(P10*). The healthcare providers’ approach to shared decision-making was consistently seen as an important feature of excellent care by participants, with effective practitioners “[presenting] different options, saying what they would do then ultimately let patients choose” *(P24*). This collaborative approach facilitated the development of what 1 participant described as a “mutual understanding” *(P26)* of therapeutic choices.

The management of therapeutic information was a key mechanism through which patients exercised agency, though information needs evolved throughout the treatment journey. Initial encounters with detailed therapeutic information “could be overwhelming” *(P1)*, with participants expressing preference for more detailed information further in the management journey: “what would be more useful is an overview of what the steps are, because there’s a fear of the unknown” *(P19)*. Key informational priorities included understanding risk profiles, fertility implications—particularly “information on becoming pregnant on a drug” *(P18)—*and therapeutic efficacy, while demonstrating sophisticated understanding of treatment variability, acknowledging individual variation in treatment response: “what works for someone else might not work for [them]” *(P11)*. Knowledge of non-drug aspects of management, including nutritional guidance, supported self-management strategies through avoidance of “unhealthy eating” *(P24)*. Furthermore, participants emphasized how knowledge of potential future therapeutic options enhanced their sense of control and maintaining agency in ongoing disease management.

## Discussion

This qualitative study provides novel insights into considerations made by patients with moderate-to-severe IBD regarding advanced therapies. The 4 themes developed through reflexive thematic analysis—embedding treatment realities into daily routines; negotiating the tension between therapeutic benefits and potential risks; navigating formal and informal support structures; and asserting agency in treatment decisions—demonstrate the complex interplay between clinical needs and lived experience that shapes patient decision-making regarding advanced therapies. Previous research^12–18^^,^^24^ identifies how factors such as route of administration, efficacy and side-effects are important, however our findings show how these priorities interact amongst participants who demonstrated sophisticated understandings of risk-benefit tradeoffs between contrasting aspects of their care. Consistent with existing literature, participants prioritized effective symptom control and treatment practicality.^24–26^ Unlike previous research suggesting dominant safety concerns about drugs from patients,^26^ our study suggests a more pragmatic approach to risk assessment by patients, prioritizing disease control while acknowledging safety considerations.

While discrete choice experiments (DCEs)^12^^,^^18^ have provided valuable quantitative insights into the relative importance of treatment attributes, our qualitative exploration reveals the underlying mechanisms and contextual factors that drive these preferences. DCE studies consistently demonstrate that patients prioritize efficacy and safety attributes. Our findings go further, illuminating how these priorities are dynamically negotiated within the broader context of patients’ lives and life experiences. A particularly novel aspect of our findings is the identification of temporal shifts in patient priorities depending on disease activity and treatment experience, suggesting that treatment decision-making is a fluid, contextual process rather than a fixed utility calculation. During active disease phases, participants prioritized rapid symptom control and were willing to accept greater inconvenience or risk.

Furthermore, while DCEs excel at quantifying tradeoffs between predetermined attributes, our thematic analysis reveals how patients construct meaning around treatment decisions through complex interactions between practical constraints, support networks, and agency in care. These factors that are often unmeasured in structured choice experiments. Indeed, the physical and social implications of treatment were prominent findings in participants’ interviews. Treatment integration into daily routines encompassed not just the practicalities of drug administration but broader impact on biographical continuity—maintaining employment, social relationships, and future planning. This extends beyond simple convenience to reflect how patients situate therapeutic decisions within their wider life context.

The findings reinforce the need for healthcare systems to evolve beyond traditional metrics of treatment success and better align therapeutic and monitoring approaches with patients’ lived experiences of managing IBD^27^ by considering how methods of drug administration, disease monitoring and follow-up support or impede patient autonomy. Advances in IBD care over the last decade have emphasized treat-to-target strategies^28^ and therapeutic drug monitoring,^29^ with STRIDE guidelines highlighting the importance of both objective disease control and patient-reported outcomes.^30^ Yet, our findings suggest the need to integrate these approaches more flexibly with patient preferences and daily routines.

The rise in remote consultation and monitoring during the COVID-19 pandemic^31^ has demonstrated the feasibility of flexible consultation modalities, offering remote follow-up during periods of disease stability while maintaining access to face-to-face care during flares or treatment transitions. The expressed preference for noninvasive monitoring strategies, such as fecal calprotectin over endoscopic assessment, reflects how patients actively negotiate the balance between therapeutic necessity and minimizing disruption to daily routines.

While oral and subcutaneous therapies were often preferred for their convenience, participants demonstrated pragmatic decision-making by expressing willingness to accept infusion-based treatments if these offered optimal disease control, particularly during periods of active disease. This pragmatic approach mirrors the evolving therapeutic landscape in IBD, where treatment choices have expanded beyond traditional step-up approaches to more personalized strategies based on individual disease characteristics and patient factors.^32^

The temporal nature of information needs, based on disease activity is an important finding, relevant for IBD services. This finding suggests the need for adaptive information provision strategies that acknowledge patients’ changing capacity to process and integrate complex therapeutic information throughout their disease course. Another novelty is the selective engagement with peer support which challenges assumptions about universal benefit of community support.^33^^,^^34^

Here, we identified strategic approaches to community/peer support, sometimes deliberately limiting exposure to others’ illness narratives. While online forums provided valuable support for some, others actively avoided such spaces. This suggests the need for healthcare providers to offer and signpost to varied support mechanisms rather than assuming traditional peer support models meet all patients’ needs. Importantly, the findings further highlight how treatment related preferences are not fixed but are rather dynamically influenced by disease activity, therapeutic response, and individual life circumstances—a reality that modern IBD care pathways must acknowledge and accommodate to achieve both clinical and patient-reported treatment targets.

**Figure 1:**
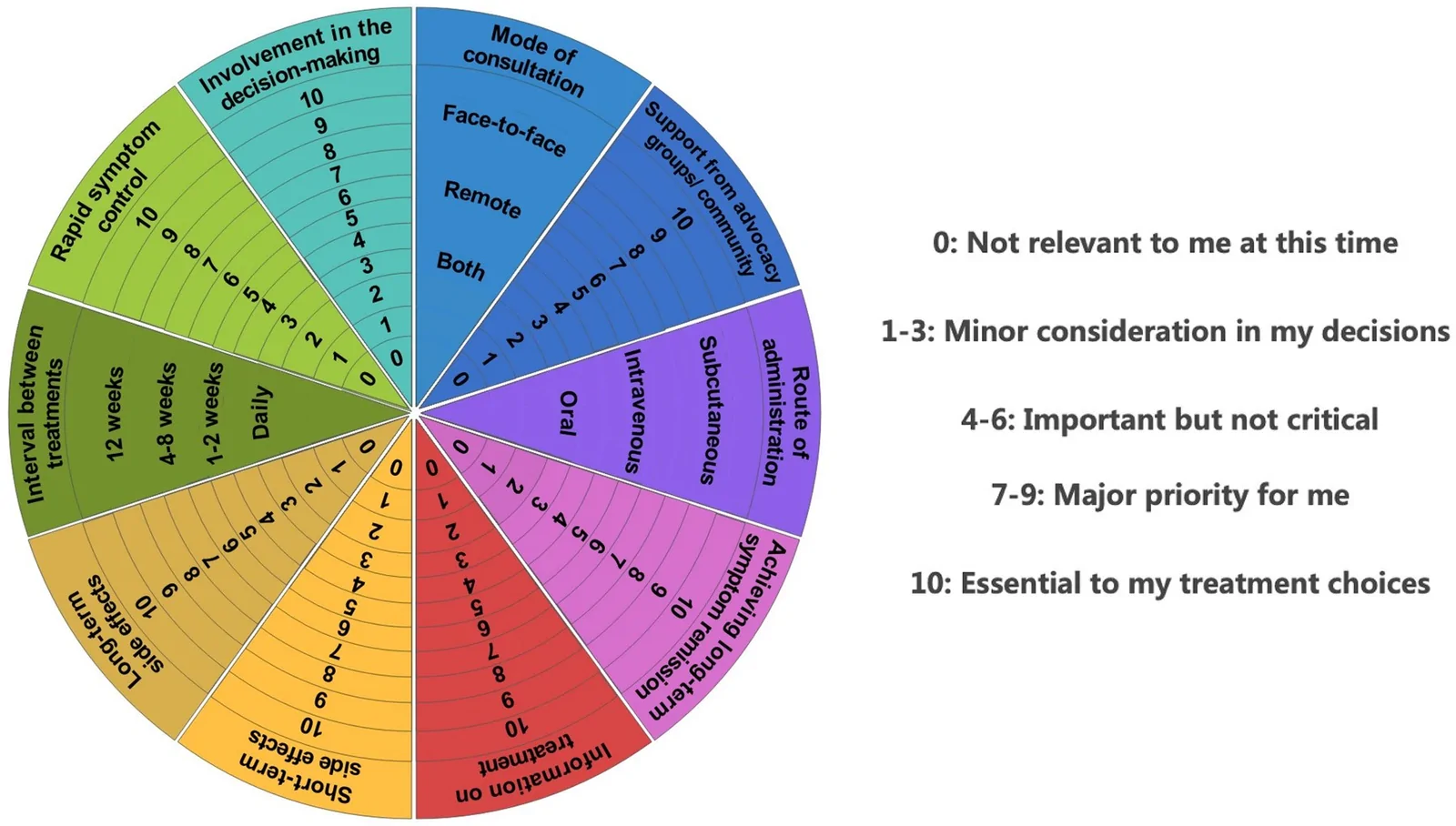
ADVOCATE-IBD tool.

The findings of this study informed the development of the advanced therapy options, choice, access, and treatment empowerment for inflammatory bowel disease (ADVOCATE-IBD) Tool, shown in Figure 1, modeled after the IBD Disk.^23^ It captures ten domains derived from our qualitative analysis that participants consider important when making decisions about advanced therapies for the management of moderate-to-severe IBD. It includes 7 measurable domains: involvement in therapeutic decision-making, support from advocacy groups/community, achieving long-term symptom remission, information on treatment, short-term side effects, long-term side effects, and achieving rapid symptom control, rated 0-10 by patients based on their priorities at that particular point in time, and 3 domains with discrete options: mode of consultation, interval between treatments, and route of administration. The ADVOCATE-IBD Tool builds upon the validated framework of the IBD Disk^23^ but extends beyond functional assessment to capture treatment-specific priorities. Its implementation could enhance routine clinical consultations by providing a preliminary structured framework for eliciting patient preferences before therapeutic decisions are made. It has potential to allow clinicians to develop a foundational understanding of individual values and priorities that can guide consultations in a tailored and efficient manner.

The tool’s design also reflects the dynamic nature of patient preferences revealed in our study, providing structure for meaningful dialogue about treatment choices while maintaining flexibility for individual circumstances. The tool’s utility extends beyond individual consultations. At a service level, systematic collection of patient preferences through ADVOCATE-IBD could inform service design and resource allocation. In practice, operationalization of the tool could involve brief pre-consultation completion by patients, with clinicians using the resulting priority profile to direct discussion toward the domains of highest relevance; for example, a patient who scores ‘long-term side effects’ as a high priority might benefit from a targeted conversation focused on the safety profile and monitoring plan for their chosen therapy, whilst a patient who prioritizes ‘rapid symptom control’ may prompt early discussion of induction response timelines and dose escalation criteria.

The study had a few limitations. First, participants were recruited exclusively through voluntary self-selection using social media, patient advocacy groups and clinicians directing patients to online information for them to self-refer, potentially leading to selection bias toward participants who are more actively engaged with their healthcare and comfortable discussing their experiences. Second, the sample demographics showed limited ethnic diversity, with most participants being white British, potentially reducing transferability of findings to other ethnic groups where cultural factors may influence treatment experiences and decision-making. Third, although all participants were in clinical remission at the time of interview, objective disease activity was not prospectively recorded using validated endoscopic or biochemical measures. Fourth, while virtual interviews enabled broad geographical recruitment, the lack of face-to-face interaction may have impacted rapport-building and reduced the richness of non-verbal communication data. Fifth, while we included patients’ on different advanced therapies reflecting the prescribing landscape at the time of recruitment, other newer advanced therapies, including selective interleukin-23 (IL-23) inhibitors and sphingosine-1-phosphate (S1P) receptor modulators, have since received NICE approval either shortly before or during the recruitment period and had not yet entered mainstream prescribing practice. Consequently, no participants were receiving these agents, and patient perspectives specific to these therapies could not be captured in the current dataset.

Finally, while our 26 participants represented diverse geographical locations across the United Kingdom, we recognize that individual participants cannot fully represent the heterogeneity of experiences within their respective regions. Participants received care across various healthcare settings including district general hospitals, teaching hospitals, and tertiary centers. However, analysis of differences in perspectives based on facility type was beyond the scope of this qualitative research. Regarding sample size, 26 participants is consistent with established principles of qualitative inquiry, where adequacy is determined by data saturation^21^ rather than statistical power. Future research should aim to refine the ADVOCATE-IBD tool based on findings from non-UK healthcare settings, involving a diverse population. Moreover, evaluation of the tool’s clinical impact and acceptability, including its effect on decisional conflict, patient satisfaction, and quality of consultations, represents another important direction for future research; a formal implementation study, would provide the methodological structure to assess these outcomes in a real-world setting and include participants on the newly approved agents.

## Conclusion

This qualitative study demonstrates how patient decision-making around advanced therapies for management of moderate-to-severe IBD is shaped by the complex interplay between treatment practicality, therapeutic efficacy, risk management, support structures and healthcare engagement. Patients actively construct their treatment preferences through ongoing negotiation of practical constraints, disease burden, and desired life participation, while priorities differ depending on acuity of disease. The findings revealed sophisticated decision-making processes that balance immediate disease control against long-term considerations. Healthcare delivery must therefore evolve beyond conventional metrics of treatment success and standard protocolised approaches to develop a nuanced understanding of individual patient priorities when making decisions about advanced therapy selection, acknowledging this complexity and supporting patient agency in therapeutic choices.

Drawing upon these qualitative insights, we developed the ADVOCATE-IBD framework as a systematic tool for capturing patient preferences in clinical practice. This framework provides clinicians with a structured approach to understanding individual patient values and priorities that can guide shared decision-making consultations. While ready for clinical deployment, the tool requires further evaluation and context-specific refinement through real-world application across diverse healthcare systems to assess its utility, acceptability, and effectiveness in enhancing patient-centered care within the expanding therapeutic landscape of IBD management.

## Supplementary Material

otag098_Supplementary_Data

## Data Availability

Data are available upon reasonable request.
