# Supplementary material for: Advanced therapy options, choice, access, and treatment empowerment for inflammatory bowel disease (ADVOCATE-IBD): a UK-wide semi-structured interview study exploring patient preferences around advanced therapy decisions in inflammatory bowel disease
Source: Crohns Colitis 360. 2026 Aug 28;8(3):otag098. doi: 10.1093/crocol/otag098 (PMC13588702; doi:10.1093/crocol/otag098)
Supplement: Supplementary file 1 [file otag098_supplementary_data.docx]

**Appendices**

**Appendix 1: Topic guide**

**Introduction**

- Introduce self
- Thank Participant
- Explain purpose to understand what matters to them when being on an advanced therapy
- Emphasise importance of speaking clearly, not too fast to help transcription

**Participant and drug-related**

- What age range are you in? (18-20, 21-30, 31-40, 41-50, 51-60, 61-70, 71-80, 80+)
- What is your gender?
- Which condition do you suffer from? Crohn’s or UC?
- How long ago were you diagnosed?
- Which region (city/ Town) do you receive your IBD care in?
- Which biological drug are you on now and how you have it (orally/ injection/ infusion)?
- How long have you been on the latest biological drug for?
- Which ones were you on before?
  - How would you rank these?
  - Why?
- Walk me through when you were first started on biologics/ advanced therapies?
  - What were your priorities at the time?
- How did you and your IBD team decide which drug was right for you?
  - What mattered most in that decision?
  - How involved were you in the decision-making when starting/ changing drug?
- What were your hopes or concerns when beginning the medication?
- Now that you’ve been on an advanced therapy for some time, what would you say matters most to you regarding your IBD care?
- How important is it to have an effective biologic that keeps your IBD symptoms under control?
  - Why?
- How would you describe your day-to-day experiences of being on long-term advanced therapy?
- How do medication side effects impact your treatment experiences and priorities?
- What role do dosing frequency and administration route of your biological play in your preferences?
- Rank the following modes of delivery for your IBD drug in order of preference (**Explore why**)
  - Oral
  - Subcutaneous (self-administered)
  - IV Infusion (in hospital)

**Monitoring**

- How often do you need to go for monitoring on therapy?
  - Explore views on monitoring
- How often do you see your IBD nurse?
  - Explore views on care received by IBD nurse
- How often do you see your IBD specialist?
  - Explore views on care received

**Service**

- How important is it to hear from other patients or see them in person?
- How important is it to receive dietary advice or see a dietician?
- How important is the frequency of monitoring tests and appointments to you while on biologics?
- Do you have a preference between in-person vs virtual care while on advanced therapy? Why?
- How much does continuity with the same care providers matter to you?
- What value do you place on being involved in decisions about your treatment changes?
- Are there any attributes or practices from your healthcare practitioners that help promote shared decision making?
- How would you describe your relationship with your care provider and the level of trust between you?
- What are the financial costs associated with being on an advanced therapy in your eyes?
- How empowered and confident do you feel managing your illness while on advanced therapies?

**Concluding comments**

- If you could change one thing about your IBD care while on biologics, what would have the biggest impact on your quality of life?
- Any final perspectives to share on optimising biologics treatment for patient priorities

**Ending**

- Thank them for time and contribution to study
- Confirm how their interview data will be used

**Appendix 2: SRQR Checklist**

|  | **Standards for Reporting Qualitative Research (SRQR)*** |  |
| --- | --- | --- |
|  | <http://www.equator-network.org/reporting-guidelines/srqr/> |  |
|  |  | **Page/line no(s).** |
| **Title and abstract** | |  |
|  | **Title** - Concise description of the nature and topic of the study Identifying the study as qualitative or indicating the approach (e.g., ethnography, grounded theory) or data collection methods (e.g., interview, focus group) is recommended | 2 |
|  | **Abstract** - Summary of key elements of the study using the abstract format of the intended publication; typically includes background, purpose, methods, results, and conclusions | 2 |
|  |  |  |
| **Introduction** | |  |
|  | **Problem formulation** - Description and significance of the problem/phenomenon studied; review of relevant theory and empirical work; problem statement | 3-4 |
|  | **Purpose or research questio**n - Purpose of the study and specific objectives or questions | 3-4 |
|  |  |  |
| **Methods** | |  |
|  | **Qualitative approach and research paradigm** - Qualitative approach (e.g., ethnography, grounded theory, case study, phenomenology, narrative research) and guiding theory if appropriate; identifying the research paradigm (e.g., postpositivist, constructivist/ interpretivist) is also recommended; rationale** | 6-7 |
|  | **Researcher characteristics and reflexivity** - Researchers’ characteristics that may influence the research, including personal attributes, qualifications/experience, relationship with participants, assumptions, and/or presuppositions; potential or actual interaction between researchers’ characteristics and the research questions, approach, methods, results, and/or transferability | 6-7 |
|  | **Context** - Setting/site and salient contextual factors; rationale** | 6 |
|  | **Sampling strategy** - How and why research participants, documents, or events were selected; criteria for deciding when no further sampling was necessary (e.g., sampling saturation); rationale** | 4-5 |
|  | **Ethical issues pertaining to human subjects** - Documentation of approval by an appropriate ethics review board and participant consent, or explanation for lack thereof; other confidentiality and data security issues | 5-6 |
|  | **Data collection methods** - Types of data collected; details of data collection procedures including (as appropriate) start and stop dates of data collection and analysis, iterative process, triangulation of sources/methods, and modification of procedures in response to evolving study findings; rationale** | 6 |
|  | **Data collection instruments and technologies** - Description of instruments (e.g., interview guides, questionnaires) and devices (e.g., audio recorders) used for data collection; if/how the instrument(s) changed over the course of the study | 6 |
|  | **Units of study** - Number and relevant characteristics of participants, documents, or events included in the study; level of participation (could be reported in results) | 7-8 |
|  | **Data processing** - Methods for processing data prior to and during analysis, including transcription, data entry, data management and security, verification of data integrity, data coding, and anonymization/de-identification of excerpts | 6 |
|  | **Data analysis** - Process by which inferences, themes, etc., were identified and developed, including the researchers involved in data analysis; usually references a specific paradigm or approach; rationale** | 6 |
|  | **Techniques to enhance trustworthiness** - Techniques to enhance trustworthiness and credibility of data analysis (e.g., member checking, audit trail, triangulation); rationale** | 6-7 |
|  |  |  |
| **Results/findings** | |  |
|  | **Synthesis and interpretation** - Main findings (e.g., interpretations, inferences, and themes); might include development of a theory or model, or integration with prior research or theory | 7-13 |
|  | **Links to empirical data** - Evidence (e.g., quotes, field notes, text excerpts, photographs) to substantiate analytic findings | 7-13 |
|  |  |  |
| **Discussion** | |  |
|  | **Integration with prior work, implications, transferability, and contribution(s) to the field -** Short summary of main findings; explanation of how findings and conclusions connect to, support, elaborate on, or challenge conclusions of earlier scholarship; discussion of scope of application/generalizability; identification of unique contribution(s) to scholarship in a discipline or field | 14-19 |
|  | **Limitations** - Trustworthiness and limitations of findings | 18-19 |
|  |  |  |
| **Other** | |  |
|  | **Conflicts of interest** - Potential sources of influence or perceived influence on study conduct and conclusions; how these were managed | Submitted directly to journal |
|  | **Funding** - Sources of funding and other support; role of funders in data collection, interpretation, and reporting | Submitted directly to journal |
|  |  |  |
|  | *The authors created the SRQR by searching the literature to identify guidelines, reporting standards, and critical appraisal criteria for qualitative research; reviewing the reference lists of retrieved sources; and contacting experts to gain feedback. The SRQR aims to improve the transparency of all aspects of qualitative research by providing clear standards for reporting qualitative research. |  |
|  |  |  |
|  | **The rationale should briefly discuss the justification for choosing that theory, approach, method, or technique rather than other options available, the assumptions and limitations implicit in those choices, and how those choices influence study conclusions and transferability. As appropriate, the rationale for several items might be discussed together. |  |
|  |  |  |
|  | **Reference:** |  |
|  | O'Brien BC, Harris IB, Beckman TJ, Reed DA, Cook DA. **Standards for reporting qualitative research: a synthesis of recommendations.** *Academic Medicine*, Vol. 89, No. 9 / Sept 2014  DOI: 10.1097/ACM.0000000000000388 |  |
|  |  |  |
|  |  |  |
